# Cannabinoid Receptor Type 1 (CB1R) Expression in Limbic Brain Structures After Acute and Chronic Seizures in a Genetic Model of Epilepsy

**DOI:** 10.3389/fnbeh.2020.602258

**Published:** 2020-12-21

**Authors:** Willian Lazarini-Lopes, Rui M. P. da Silva-Júnior, Gabriel Servilha-Menezes, Raquel A. Do Val-da Silva, Norberto Garcia-Cairasco

**Affiliations:** ^1^Neuroscience and Behavioral Sciences Department, Ribeirão Preto School of Medicine, University of São Paulo, Ribeirão Preto, Brazil; ^2^Neurophysiology and Experimental Neuroethology Laboratory, Physiology Department, Ribeirão Preto School of Medicine, University of São Paulo, Ribeirão Preto, Brazil; ^3^Department of Internal Medicine, Ribeirão Preto School of Medicine, University of São Paulo, Ribeirão Preto, Brazil

**Keywords:** CB1R expression, WAR strain, audiogenic seizures, amygdala, hippocampus

## Abstract

The endocannabinoid system (ECS) is related to several physiological processes, associated to the modulation of brain excitability, with impact in the expression of susceptibility and control of epileptic seizures. The cannabinoid receptor type 1 (CB1R) is widely expressed in the brain, especially in forebrain limbic structures. Changes in CB1R expression are associated with epileptic seizures in animal models and humans. The Wistar Audiogenic Rat (WAR) strain is a genetic model of epilepsy capable of mimicking tonic-clonic and limbic seizures in response to intense sound stimulation. The WAR strain presents several behavioral and physiological alterations associated with seizure susceptibility, but the ECS has never been explored in this strain. Therefore, the aim of the present study was to characterize CB1R expression in forebrain limbic structures important to limbic seizure expression in WARs. We used a detailed anatomical analysis to assess the effects of acute and chronic audiogenic seizures on CB1R expression in several layers and regions of hippocampus and amygdala. WARs showed increased CB1R immunostaining in the inner molecular layer of the hippocampus, when compared to control Wistar rats. Acute and chronic audiogenic seizures increased CB1R immunostaining in several regions of the dorsal hippocampus and amygdala of WARs. Also, changes in CB1R expression in the amygdala, but not in the hippocampus, were associated with limbic recruitment and limbic seizure severity in WARs. Our results suggest that endogenous alterations in CB1R immunostaining in WARs could be associated with genetic susceptibility to audiogenic seizures. We also demonstrated CB1R neuroplastic changes associated with acute and chronic seizures in the amygdala and hippocampus. Moreover, the present study brings important information regarding CB1R and seizure susceptibility in a genetic model of seizures and supports the relationship between ECS and epilepsy.

## Introduction

The endocannabinoid system (ECS) is an important mechanism of biological signaling related to several physiological functions throughout the entire body (Ruiz de Azua and Lutz, 2019). In the epilepsy research scenario, the ECS has been associated with epileptic seizure susceptibility in both preclinical models of epilepsies and humans (Alger, 2004; Rosenberg et al., 2017; Lazarini-Lopes et al., 2020). Classically, the ECS comprises two G-protein-coupled receptors, the cannabinoid receptors type 1 (CB1R) and type 2 (CB2R), their endogenous ligands, called endocannabinoids (eCBs), anandamide and 2-arachidonoylglycerol (2-AG), besides the enzymes and proteins responsible for their synthesis, degradation, and transportation (Freund et al., 2003; Castillo et al., 2012). In the brain, the eCBs are synthetized “on-demand” at postsynaptic terminals and then activate CB1R located at presynaptic terminals, inhibiting neuronal firing (Lutz, 2004; Katona and Freund, 2008; Araque et al., 2017).

The CB1R is a Gi/o protein-coupled receptor widely expressed in the brain, especially in areas such as cerebellum, cortex, substantia nigra, and limbic structures (Herkenham et al., 1990; Tsou et al., 1998). Generally, CB1R regulate glutamatergic and GABAergic neurotransmission in presynaptic terminals (Katona et al., 2001; Hill et al., 2007; Turu and Hunyady, 2010), although postsynaptic CB1R modulation was also detected in the brain (Busquets-Garcia et al., 2018). CB1R are associated with epilepsies and several epilepsy-related comorbidities, such as anxiety, depression, and autism (Bhattacharyya et al., 2017; Hosie et al., 2018; Rocha et al., 2020). Additionally, changes in CB1R expression were observed in limbic brain sites after limbic seizures in animal models and humans with epilepsies (Wallace et al., 2003; Falenski et al., 2009; Maglóczky et al., 2010; Rocha et al., 2020). However, the effects of acute (brainstem) and chronic (limbic) audiogenic seizures on CB1R expression are unknown. Therefore, the role behind CB1R expression in preclinical models of epilepsies still needs to be explored.

The Wistar Audiogenic Rat (WAR) strain is a genetic model of epilepsy in which animals are capable of developing audiogenic seizures (AS) in response to intense sound stimulation (110–120 dB) (Garcia-Cairasco et al., 2017). The acute AS are modulated by brainstem sites and characterized by tonic-clonic behaviors (Garcia-Cairasco, 2002; Raisinghani and Faingold, 2003). However, during the chronic protocol of acoustic stimulation, the audiogenic kindling (AuK) (Marescaux et al., 1987), WARs, similar to Genetically epileptic-prone rats (GEPRs) (Naritoku et al., 1992) can develop limbic seizures dependent on forebrain limbic structures, such as the hippocampus and the amygdala, through an epileptogenic process called limbic recruitment (LR), with behavioral, EEG and histological correlates (Garcia-Cairasco et al., 1996; Moraes et al., 2000; Romcy-Pereira and Garcia-Cairasco, 2003; Galvis-Alonso et al., 2004). For these reasons, the AuK in WARs is considered a model of temporal lobe epilepsy (TLE) capable of mimicking limbic seizures (Moraes et al., 2000; Garcia-Cairasco et al., 2017), similar to those present in other models of TLE (Racine, 1972; Cavalheiro et al., 1991).

Although several physiological modifications have already been associated with seizure susceptibility in WARs (for a detailed review see Garcia-Cairasco et al., 2017) the ECS has never been characterized neither in WARs nor in any other audiogenic strain. Therefore, the present study was aimed to verify if the WAR strain, a genetic model of epilepsy, presents alterations in CB1R expression in the hippocampus and amygdala, some of the most important forebrain structures associated to limbic seizures expression. Additionally, we verified if acute and chronic audiogenic seizures could modulate CB1R expression in these limbic brain sites.

## Materials and Methods

### Animals and Ethical Aspects

Male WARs (*n* = 20) and Wistar (*n* = 5) rats (4 months old) were provided by the Special Rat Strain Vivarium at the Ribeirão Preto School of Medicine and by the Central Vivarium of the University of São Paulo, Ribeirão Preto, respectively. During the entire experimental protocol, animals were maintained at the Animal Housing Facility located at the Physiology Department of the Ribeirão Preto School of Medicine. Animals were kept in acrylic cages (3-4 animals/cage), in a room with controlled temperature (23 ± 2°C) and light/dark cycle of 12/12 h (lights on at 6:00 a.m.), with food and water *ad libitum*.

The experimental protocol was approved by the Ethics Committee in Animal Research of the Ribeirão Preto School of Medicine at the University of São Paulo (Protocol number: 057/2017) and all efforts were made to minimize the animal's suffering.

### Acute and Chronic Audiogenic Seizures

The protocol used to induce AS was similar to the one described in previous studies with WARs (Garcia-Cairasco et al., 1996; Moraes et al., 2000; Galvis-Alonso et al., 2004). Briefly, animals were placed into an acrylic cylindrical chamber (height: 32 cm, diameter: 30 cm) located at a soundproof chamber (45 × 45 × 40 cm). A small speaker was connected to a computer and placed on the top of the acrylic chamber. The sound (110–120 dB; 5–20 kHz) was manually triggered by the researcher and applied until the appearance of a tonic seizure, or for a maximum of 60 s.

To assess the effects of an acute brainstem (tonic-clonic) audiogenic seizure on CB1R expression, WARs were submitted to a single acoustic stimulation (WAR AS; *n* = 5). The AuK protocol was applied to investigate the effects of chronic seizures on CB1R expression in WARs (WAR AuK; *n* = 10). In the present study, 20 acoustic stimulations were applied during 10 days, every morning (8–9 a.m.) and afternoon (5–6 p.m.). Animal behavior was recorded in every acoustic stimulation for behavioral analysis. Control groups were composed by WARs submitted to the sham protocol of acoustic stimulation (with no sound) to assess the endogenous expression of CB1R in WARs (*n* = 5). Likewise, Wistar control rats (*n* = 5) were submitted to the sham protocol to access CB1R in a control non-epileptic strain.

### Behavioral Analysis

The Categorized Severity Index (CSI) (Rossetti et al., 2006) and the Racine's scale (Racine, 1972) were used to measure brainstem and limbic seizure severity, respectively, in every acoustic stimulation (Table 1).

**Table 1:** Behavioral indexes used to analyze audiogenic seizures.

**Categorized Severity Index (CSI)—Tonic-clonic seizures**		**Racine's scale—Limbic seizures**	
0	No seizure	0	No seizure
1	One running	1	Facial and ears myoclonus
2	One wild running (running with jumping and atonic fall)	2	Head myoclonus
3	Two wild runnings	3	Forelimb myoclonus
4	Tonic convulsion	4	Forelimb myoclonus followed by elevation
5	Tonic seizures followed by generalized clonic convulsions	5	Forelimb myoclonus followed by elevation and fall
6	Head ventral flexion plus CSI 5		
7	Forelimb hyperextension plus CSI 6		
8	Hindlimb hyperextension plus CSI 7		

*Categorized Severity Index used to analyze tonic-clonic seizures (Rossetti et al., 2006) and the Racine's scale used to analyze limbic seizure (Racine, 1972)*.

In the AuK protocol, behavioral criteria were applied to select animals that presented LR, the main feature of the AuK protocol (Naritoku et al., 1992; Garcia-Cairasco et al., 1996; Moraes et al., 2000). WARs that developed consistent (at least 3) and severe (Racine's scale 4–5) limbic seizures, were classified as WARs with LR. Otherwise, WARs that did not meet this criterion were classified as WAR with no limbic recruitment (NLR).

### Tissue Processing and Immunohistochemistry

Twenty-four hours after the end of the protocol, animals were anesthetized with sodium thiopental (50 mg/kg; i.p.; Abbott, Brazil) and transcardially perfused with buffer (0.1 M phosphate buffered saline, pH 7.4, 350 ml), followed by 4% paraformaldehyde (pH 7.4, 350 ml) at room temperature. The brains were removed immediately after perfusion and post-fixed in 4% paraformaldehyde for 4 hours, then tissue was cryoprotected with sucrose solution (30%) at 4°C until sinking (48–72 h). Afterwards, brain tissue was frozen in isopentane and dry ice. Serial coronal sections (40 μm) of the dorsal hippocampus and the amygdala (−1.72 mm to −3.96) were cut according to rat brain atlas (Paxinos and Watson, 2005) on a cryostat (Microm HM-505-E, Microm International, Walldorf, Germany) at −20°C and were stored in cryoprotection solution (50% PBS, 30% ethylene glycol, 20% glycerol) at −20°C until immunohistochemical procedures.

Immunohistochemistry to CB1R was performed similar as previously described (Tsou et al., 1998; McDonald and Mascagni, 2001). Briefly, we used an antibody against the synthetic peptide MSVSTDTSAE AL, corresponding to the C terminal amino acids 461–472 of Human Cannabinoid Receptor I, Anti-Cannabinoid Receptor I Rabbit polyclonal antibody (1:1,000; Ab23703, lot. GR3239384-2, Abcam). For immunohistochemistry, brain sections were washed (five times) in PBS buffer (pH 7.4), permeabilized with Triton X-100 0.3% v/v (20 min) and placed into 0.1 M glycine (15 min). After washing in PBS, endogenous peroxidase activity was blocked in 2% H_2_O_2_ solution for 30 min and the sections were incubated in block buffer (BSA 2% w/v and 0.05% Triton X-100 v/v) for 2 h at room temperature. Primary antibody, diluted in block buffer, was applied overnight at 4°C. At the following day, tissue sections were washed in PBS and then incubated for 2 h, at room temperature, with biotinylated goat anti-rabbit IgG secondary antibody (1:1,000; BA-1000, lot. Zb0318, Vector) diluted in block buffer. Finally, tissues were washed in PBS and Tris-HCl (0.05 M; pH 7.6) and the immunoreactive antigenic sites were visualized using the 3,3′-diaminobenzidine (DAB) peroxidase (HRP) substrate kit with nickel (SK-4100, Vector). Nickel was used to intensifying the DAB reaction, avoiding possible misinterpretation in regions with scarce CB1R immunostaining. Specificity of each assay was tested by omitting the primary antibody. The slices were mounted on glass slides and coverslipped with Permount (Sigma, USA).

### Image Processing and Analysis

CB1R immunostaining was assessed in Wistar and WAR rats (Wistar = 5; WAR = 5; WAR AS = 5; WAR AuK = 10). A mean of 7 sections were analyzed per animal. Images were acquired in a scanning microscope (Olympus BX61VS) with a 20x objective and all the parameters were kept the same in every image acquisition. The CB1R signal intensity was analyzed with the software imageJ (https://imagej.nih.gov/ij/) with Fiji (Schindelin et al., 2012; Schneider et al., 2012). For hippocampal layers and subregions, 3 rectangular standardized area (regions of interest, ROIs) with 2,500 μm^2^ were randomly measured in each hippocampal analyzed area. In the amygdala subnuclei, the area of each ROI was 10,000 μm^2^. The mean value of the Integrated Density (the product of area and mean gray value) was calculated using the mean value of 3 ROIs in each structure in every animal. For analysis of the total area, a manual ROI, covering the entire extension of the analyzed structure was used to assess the Integrated Density. Details of subgroup analysis are described in the Supplementary Table 1.

The heatmaps were generated using the ICY Bioimage software. The images were previously inverted and converted to gray scale, the colormap model was applied using the *Morgenstemning* colormap model as a template. The color scale bar was generated with the Color Bar 1.0.1.0 Local plugin developed by Stephane Dallongeville and available at http://icy.bioimageanalysis.org/plugin/color-bar/. The color scale bar represents the average intensity values with a variation from minimum 0 (black) to maximum 255 (white).

### Statistical Analysis

One-way ANOVA followed by a *post-hoc* Tukey's test was used when multiples groups were compared. Behavioral and immunostaining results were expressed as mean ± standard error mean (SEM). Significant values were considered when *p* < 0.05. The software Graph Pad Prism 7.0 was used to develop the statistical analysis.

## Results

### Behavioral Seizure Expression: Acute and Chronic Audiogenic Seizures

**Figure 1:**
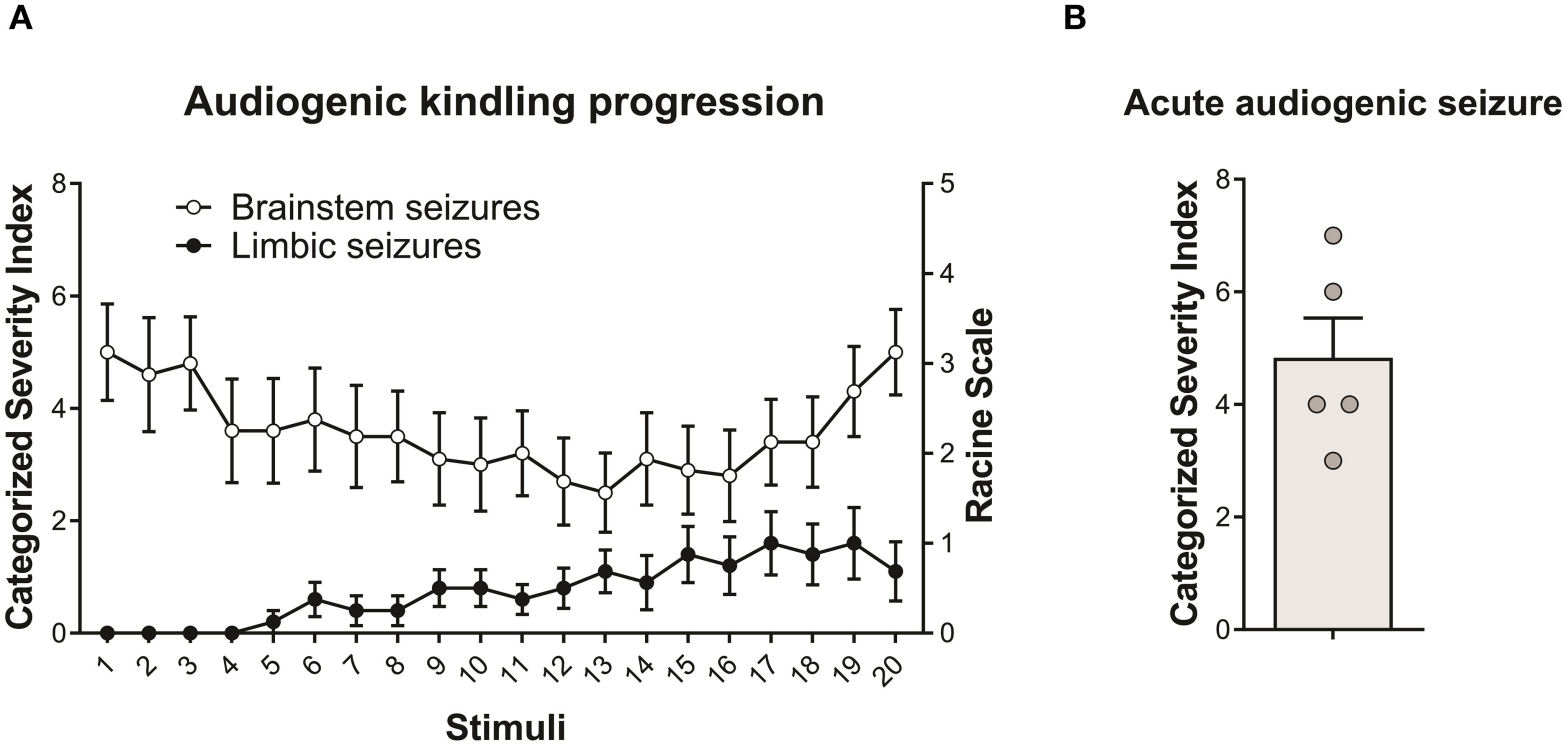
Behavioral seizure expression in Wistar Audiogenic Rats (WAR). **(A)** Evolution of audiogenic seizures in animals submitted to chronic audiogenic seizures in the audiogenic kindling (AuK) protocol (*n* = 10). The Categorized Severity Index (Rossetti et al., 2006) was used to analyze brainstem seizures, while the Racine's scale (Racine, 1972) was used to analyze limbic seizures. It is possible to observe the presence of brainstem tonic-clonic seizures (white circles) in the beginning of the protocol and the development of limbic seizures (black circles) during the chronic phase of the protocol. **(B)** Mean of brainstem seizure severity in WARs submitted to a single acoustic stimulation (*n* = 5). Data are expressed as mean ± standard error mean (SEM).

All WARs submitted to the AuK developed seizures during the protocol. Moreover, although the limbic seizures (as expected) were absent in the beginning of the AuK protocol, they became present in the chronic phase (Figure 1A). In the acute protocol of AS, 1 WAR expressed only wild running behaviors, and all the other WARs developed wild running followed by a tonic-clonic seizure (Figure 1B).

### CB1R in the Dorsal Hippocampus

**Figure 2:**
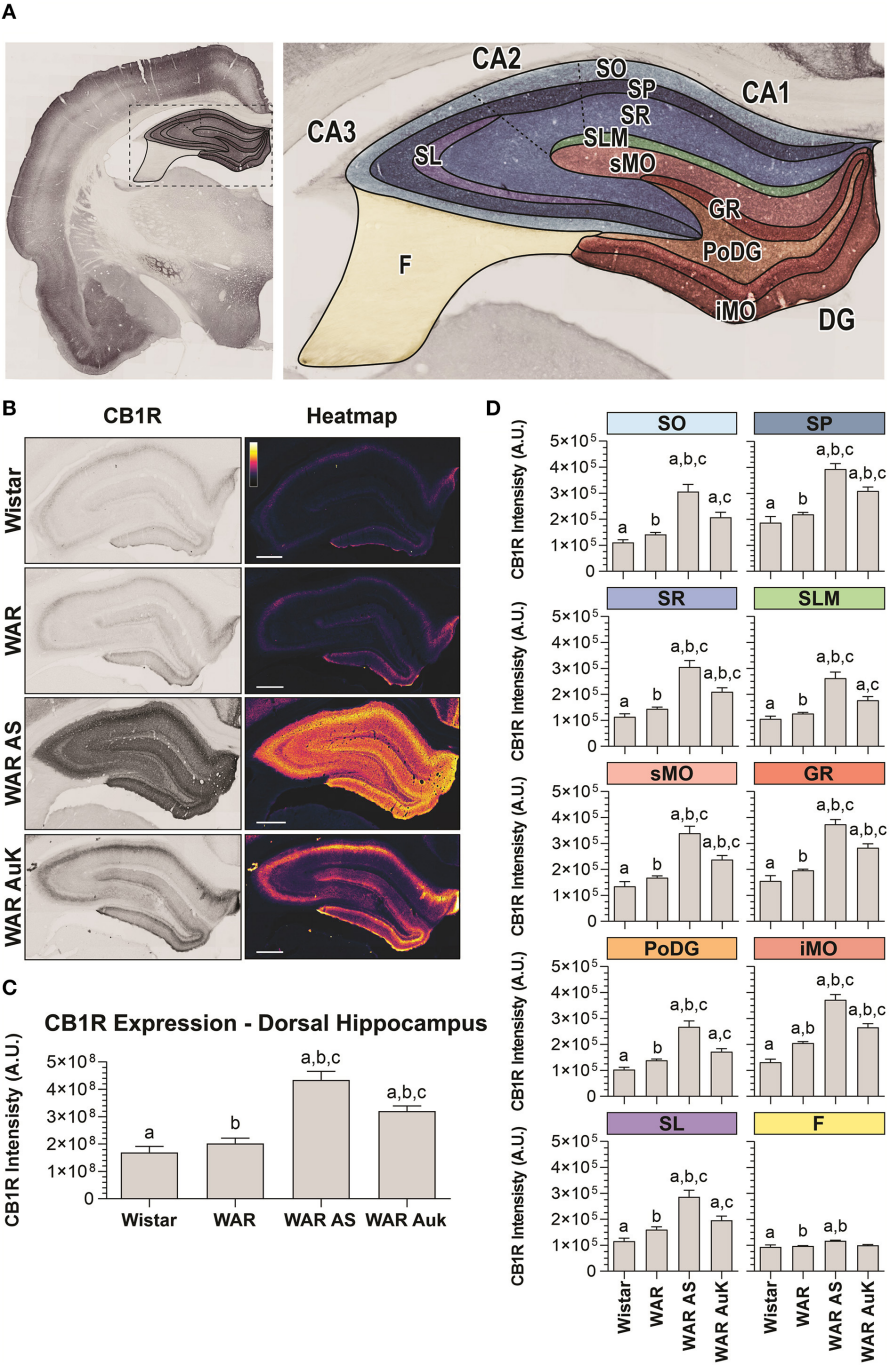
CB1R immunostaining in the hippocampus. **(A)** Schematic representation of hippocampal regions and layers used to CB1R signal quantification: Stratum oriens (SO), stratum pyramidale (SP), stratum radiatum (SR), stratum lacunosum moleculare (SLM), superior molecular layer (sMO), inferior molecular layer (iMO), dentate gyrus granular cell layer (GR), polymorph layer of the dentate gyrus (PoDG), stratum lucidum (SL), fimbria (F). **(B)** Representative images of CB1R immunostaining in the dorsal hippocampus in different experimental groups (left column) and their corresponding heatmap (right column). **(C)** Quantification of CB1R intensity in the total hippocampal area of Wistar (*n* = 5), WAR (*n* = 5), WAR AS (after acute audiogenic seizure, *n* = 5), and WAR AuK (after audiogenic kindling, *n* = 10). **(D)** CB1R intensity in different hippocampal layers of Wistar (*n* = 5), WAR (*n* = 5), WAR AS (*n* = 3), and WAR AuK (*n* = 10). Data are expressed as mean ± standard error mean (SEM). Equal letters represent significant differences (*p* < 0.05) between groups: “a” in comparison to Wistar; “b” in comparison to WAR; “c” in comparison to WAR AS. Scale bar: 500 μm. Color code scale (8 bits image): 0–255 (min–max).

Firstly, we observed significant changes between experimental groups regarding CB1R immunostaining in the total area of the dorsal hippocampus (F_(3, 21)_ = 22.41; *p* < 0.0001). *Post-hoc* analysis did not reveal endogenous differences of CB1R immunostaining in WARs, when compared to control Wistar rats (*p* > 0.05). However, the acute AS and the AuK both increased CB1R immunostaining in the total hippocampal area of WARs (*p* < 0.01), when compared to Wistar and control WARs. Curiously, the increased CB1R immunostaining was higher in WARs submitted to an acute AS than in WARs submitted to chronic (kindled) seizures (*p* < 0.01). See Figures 2A–C.

In order to verify if changes in CB1R immunostaining observed in the total hippocampal area were associated with specific regions, we analyzed several hippocampal layers: stratum oriens (SO), stratum pyramidale (SP), stratum radiatum (SR), stratum lacunosum moleculare (SLM), in the dentate gyrus, superior molecular layer (sMO), inferior molecular layer (iMO), granular cell layer (GR), polymorph layer of the dentate gyrus (PoDG), stratum lucidum (SL), and fimbria (F). See Figure 2A for anatomical details. It is important to highlight that although the term hilus became the most used in this landscape, because the evaluation of our tissue at the dentate gyrus was based upon the optical density of CB1R in layers (molecular layer, granular cell layer, polymorph layer), not in regions, the term polymorph layer is the most appropriate (Scharfman and Myers, 2013).

WARs showed increased endogenous CB1R immunostaining when compared to Wistar control rats only in the iMO layer (*p* < 0.05). Acute AS increased CB1R immunostaining in all hippocampal layers of WARs, when compared to control WARs and Wistars (*p* < 0.05). The AuK increased CB1R in all layers, except in the F, of the WAR AuK group, when compared to Wistars (*p* < 0.01), but only in the SP, SR, sMO, GR, and iMO layers (*p* < 0.05), when compared to control WARs. Moreover, WAR AS showed increased CB1R immunostaining in all hippocampal layers, except the F, when compared to WAR AuK (Figure 2D).

### CB1R in the Amygdala

**Figure 3:**
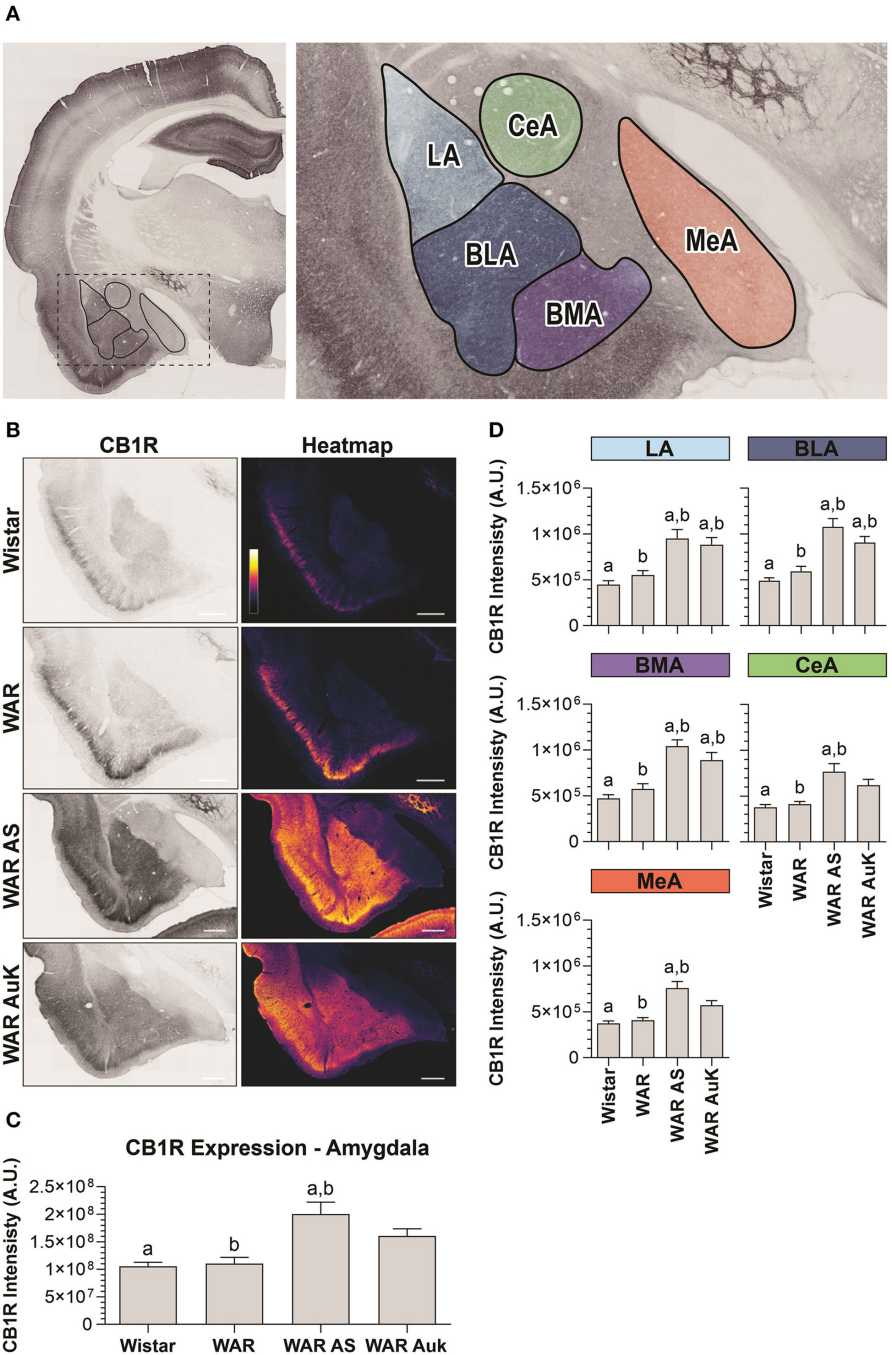
CB1R immunostaining in the amygdala. **(A)** Schematic representation of the amygdala nuclei: lateral amygdala nucleus (LA), basolateral amygdala nucleus (BLA), basomedial amygdala nucleus (BMA), central amygdala nucleus (CeA), and medial amygdala nucleus (MeA). **(B)** Representative images of CB1R immunostaining in the amygdala in different experimental groups (left column) and their correspondent heatmap (right column). **(C)** CB1R immunostaining in the total area of the amygdala of Wistar (*n* = 5), WAR (*n* = 5), WAR AS (after acute audiogenic seizure, *n* = 5) and WAR AuK (after audiogenic kindling, *n* = 10). **(D)** CB1R intensity in different amygdala nuclei of Wistar (*n* = 5), WAR (*n* = 5), WAR AS (*n* = 5) and WAR AuK (*n* = 10). Data are expressed as mean ± standard error mean (SEM). Equal letters represent significant differences (*p* < 0.05) between groups: “a” in comparison to Wistar; “b” in comparison to WAR. Scale bar: 500 μm. Color code scale (8 bits image): 0–255 (min–max).

Significant differences were observed between experimental groups regarding CB1R immunostaining in the total amygdala area [*F*_(3, 21)_ = 7.93; *p* = 0.001] (Figures 3A–C). Similarly, as occurred in the hippocampus, CB1R endogenous differences were not observed in the amygdala of WARs, when compared to control Wistars (*p* > 0.05). However, acute AS were capable of increasing CB1R immunostaining in the total area of the amygdala of WARs, when compared to Wistar and control WARs (*p* < 0.01). Additionally, it is worth to note that, although the mean of the total CB1R immunostaining in the amygdala of the WAR AuK group was higher than in control Wistar and WAR groups, these differences were not statistically significant (0.05 < *p* < 0.1). See Figure 3C.

We also analyzed CB1R immunostaining in specific amygdala nuclei: the lateral amygdala nucleus (LA), the basolateral amygdala nucleus (BLA), the basomedial amygdala nucleus (BMA), the central amygdala nucleus (CeA), and the medial amygdala nucleus (MeA). Here, it is worth to note that although the mean of endogenous CB1R signal intensity was slightly higher in all amygdaloid nuclei of control WARs in comparison to control Wistars, no statistical difference was observed (*p* > 0.05). Both acute AS and AuK were capable of increasing CB1R immunostaining in the LA, BLA, and BMA nuclei of WARs in comparison to control Wistar and WAR rats (*p* < 0.05). However, in the CeA and MeA, only the acute AS induced changes in CB1R expression, increasing CB1R immunostaining in comparison to control Wistars and WARs. No differences were observed between the WAR AS and WAR AuK groups. See Figure 3D.

### Limbic Seizures Expression in WARs With and Without Limbic Recruitment

To verify if changes in CB1R immunostaining were associated to the LR with limbic seizures in WARs, we analyzed the limbic seizure expression during the AuK, and animals were classified as WARs with LR or WARs with NLR. Six WARs developed consistent limbic seizures (Racine's scale ≥4) during the AuK and were classified as recruited (LR) WARs. From the 4 remaining WARs, 3 of them never developed limbic seizures (NLR) and 1 WAR showed 2 limbic seizures (Racine's scale 2) during the AuK. For these reasons, these rats were classified as non-recruited WARs.

**Figure 4:**
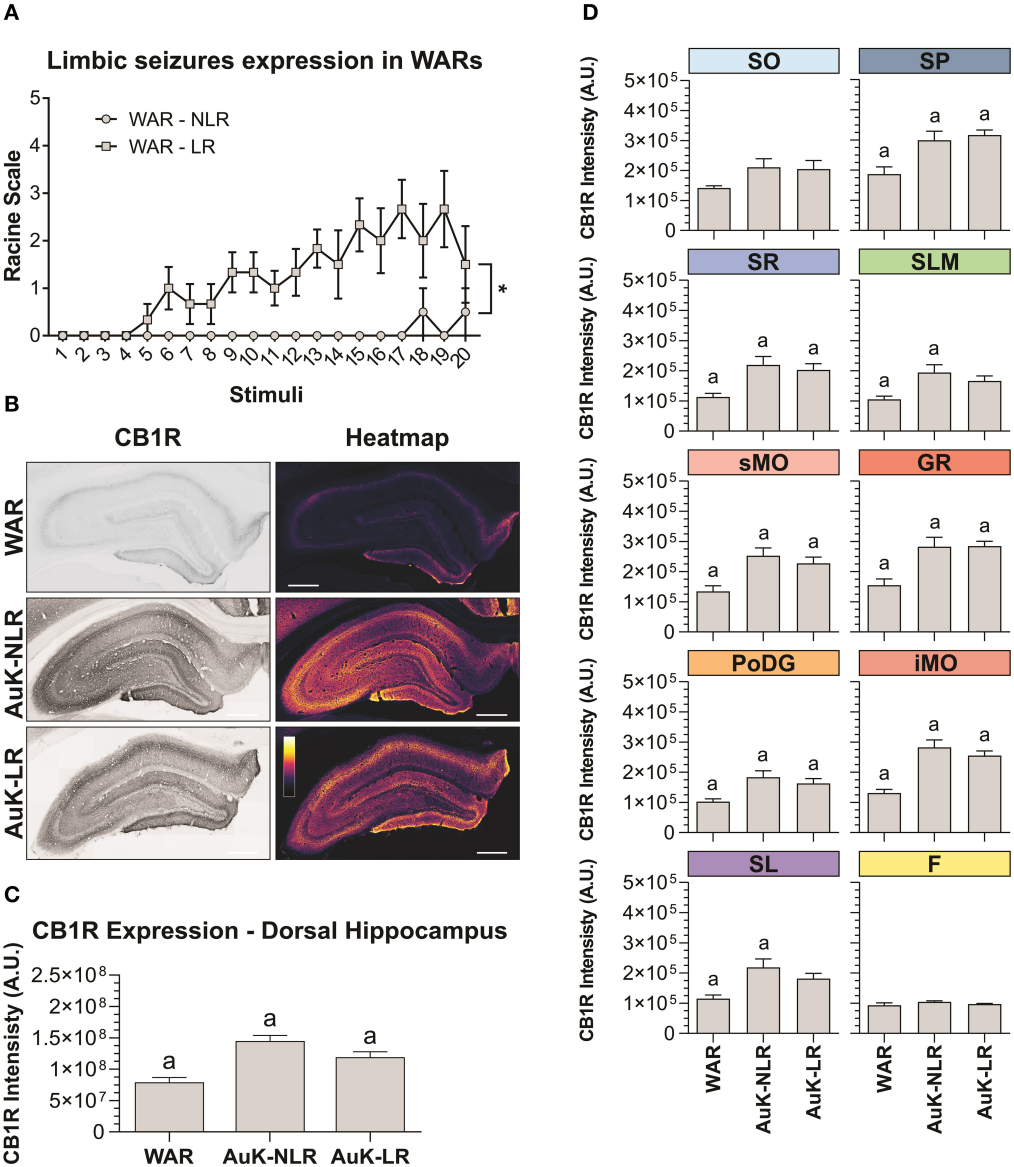
CB1R immunostaining in the hippocampus after chronic seizures: WARs with limbic recruitment (LR) and WARs with no limbic recruitment (NLR). **(A)** Limbic seizure expression in WARs during the audiogenic kindling (AuK). Squares represent limbic seizure progression in WARs with limbic recruitment (LR). Circles represent limbic seizure progression in WARs with no limbic recruitment (NLR). Seizures were analyzed according to the Racine's scale (Racine, 1972). **(B)** Representative images of CB1R immunostaining in the dorsal hippocampus in different experimental groups (left column) and their correspondent heatmap (right column). **(C)** CB1 immunostaining in the total hippocampal area of WARs (*n* = 5) compared to WARs after the AuK with limbic recruitment (LR, *n* = 6) and with no limbic recruitment (NLR, *n* = 4). **(D)** CB1R signal intensity in different hippocampal layers. Stratum oriens (SO), stratum pyramidale (SP), stratum radiatum (SR), stratum lacunosum moleculare (SLM), superior molecular layer (sMO), inferior molecular layer (iMO), dentate gyrus granular cell layer (GR), polymorph layer of the dentate gyrus (PoDG), stratum lucidum (SL), fimbria (F). Data are expressed as mean ± standard error mean (SEM). Equal letters represent significant differences (*p* < 0.05) between groups: “a” in comparison to WAR. Scale bar: 500 μm. Color code scale (8 bits image): 0–255 (min–max).

As expected, significant changes in the development of limbic seizures were observed in WARs LR compared to WARs NLR (Figure 4A). There is a statistical effect of the AuK progression (number of stimuli) on the limbic seizures expression [*F*_(19, 152)_ = 2,234; *p* = 0.0039], with limbic seizures appearing after repeated stimulations. Similarly, significant differences in limbic seizures expression were associated with the presence of LR in WARs [*F*_(1, 8)_ = 24,96; *p* = 0.0011], with recruited WARs showing increased limbic seizure frequency and severity during the AuK, when compared to non-recruited WARs (*p* < 0.05). Also, a statistical interaction between the AuK progression and limbic recruitment was observed [*F*_(19, 152)_ = 1,935; *p* = 0.0151].

### Hippocampus

Animals from the WAR AuK were divided into two different groups, WARs with LR and WARs with NLR, we observed that both groups of WARs chronically stimulated presented increased CB1R immunostaining in the total area of the dorsal hippocampus, when compared control WARs (*p* < 0.05), but no difference was observed between WAR LR and WAR NLR groups (Figures 4B,C). Several hippocampal layers and regions were analyzed to verify if changes in CB1R immunostaining in specific hippocampal sites could be associated with LR or not in WARs. WARs LR showed increased CB1 immunostaining in the SP, SR, sMO, GR, PoDG, and iMO (*p* < 0.05), while WARs NLR showed increased CB1 immunostaining in the SP, SR, SLM, sMO, GR, PoDG, iMO, and SL (*p* < 0.05). No difference was observed between WAR LR and WAR NLR in any hippocampal layer. See Figure 4D.

**Figure 5:**
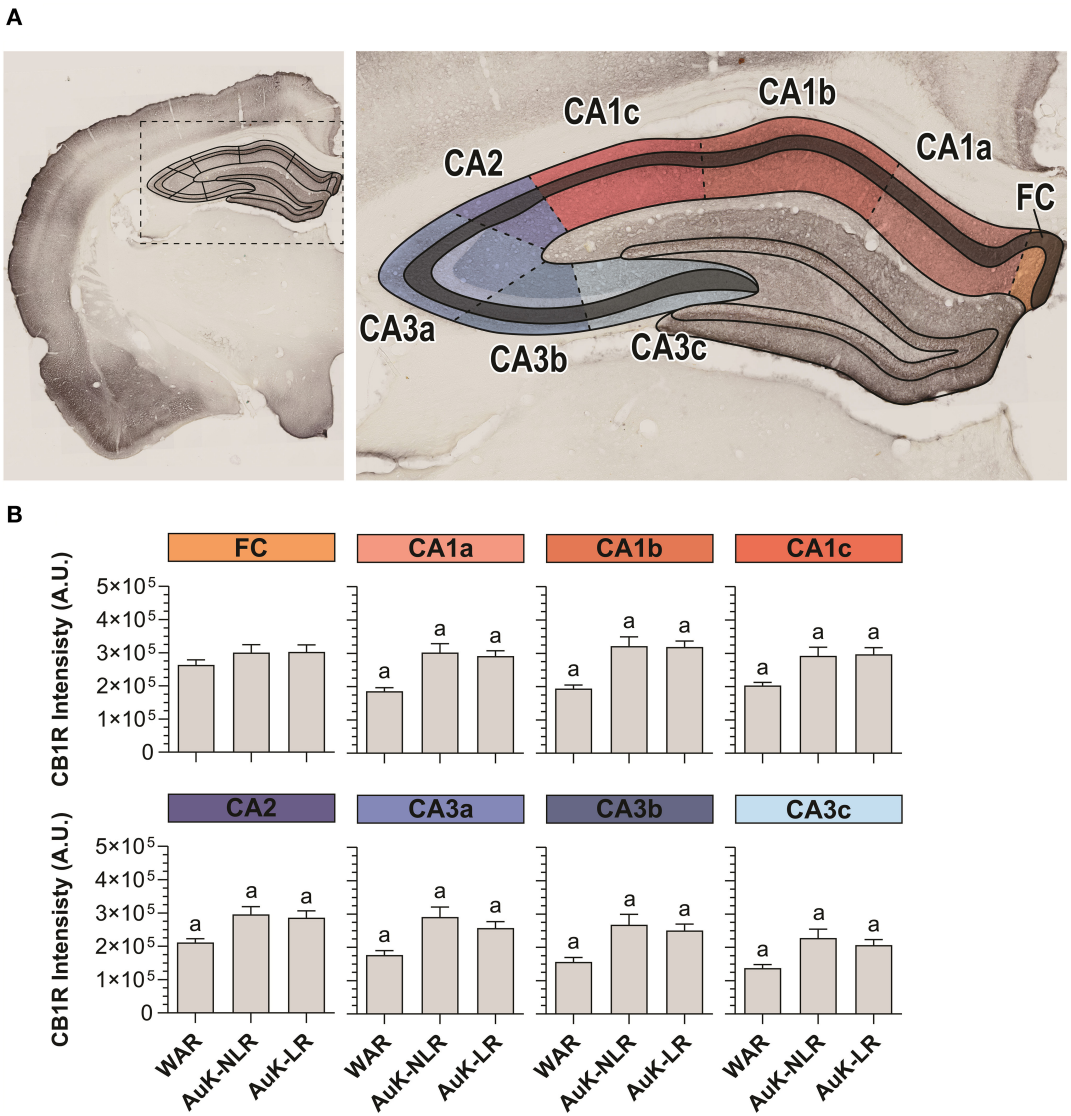
CB1R immunostaining in stratum pyramidal (SP) layer throughout the Cornu Ammonis (CA) after chronic seizures. **(A)** Schematic representation of the Cornu Ammonis area (CA1, CA2, and CA3). **(B)** CB1R immunostaining in control WARs compared to WARs with limbic recruitment (LR) and WARs with no limbic recruitment (NLR) throughout the hippocampal CA area: fasciola cinereum (FC), CA1a, CA1b, CA1c, CA2, CA3a, CA3b, and CA3c. Data are expressed by mean ± standard error mean (SEM). Equal letters represent significant differences (*p* < 0.05) between groups: “a” in comparison to WAR. Scale bar: 500 μm. Color code scale (8 bits image): 0–255 (min–max).

Hippocampal pyramidal neurons from the SP layer receive excitatory projections from several cortical areas. In their turn, hippocampal pyramidal neurons send information to dozens of other areas and subareas and they are closely associated with the epileptogenic process (Jay and Witter, 1991; Cenquizca and Swanson, 2007; Evans and Dougherty, 2018). For these reasons, we analyzed CB1R in SP throughout the Cornu Ammonis area (CA1, CA2, and CA3) of WARs submitted to the AuK (Figures 5A,B).

CA1 was divided in CA1a, CA1b, and CA1c and in all these subregions, CB1R immunostaining was increased in both, recruited and non-recruited WARs (*p* < 0.05) when compared control WARs. In the CA2, the same pattern of results was observed, with increased CB1R signal intensity in WARs NLR and WARs LR, when compared to control WARs (*p* < 0.05). Likewise, in all CA3 subregions CA3a, CA3b, and CA3c, CB1R immunostaining was increased in WARs NLR and WARs LR in comparison to control WARs (*p* < 0.05). However, no difference was observed in the fasciola cinereum (FC) of the hippocampus. Also, no difference was observed between WAR NLR and WAR LR (Figure 5B).

### Amygdala

**Figure 6:**
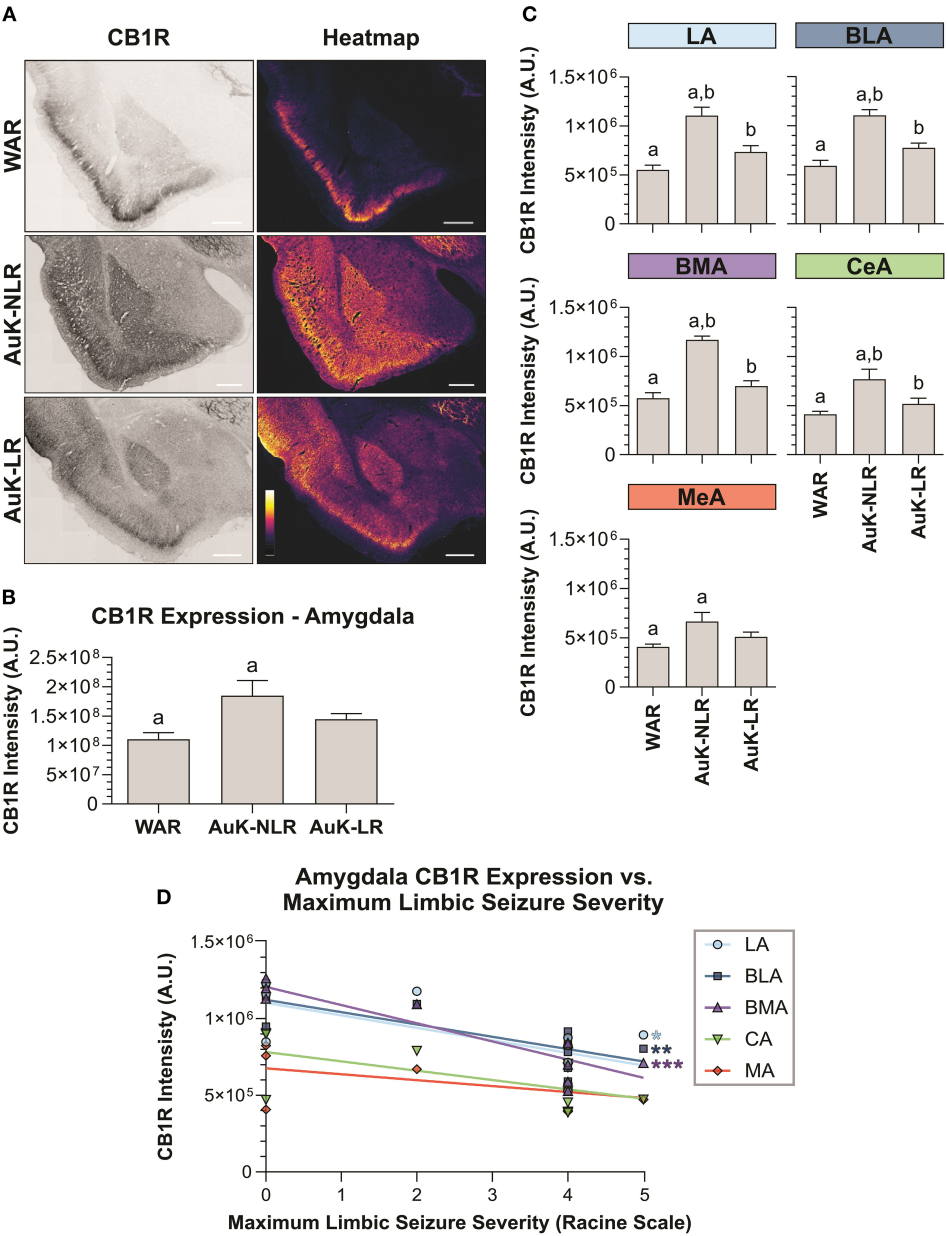
CB1R immunostaining in the amygdala after chronic seizures. WARs with limbic recruitment (LR) and WARs with no limbic recruitment (NLR). **(A)** Representative images of CB1R immunostaining in the amygdala in different experimental groups (left column) and their correspondent heatmap (right column). **(B)** CB1R immunostaining in the total amygdala area of WARs (*n* = 5) compared with WARs after the AuK with limbic recruitment (LR, *n* = 6) and with no limbic recruitment (NLR, *n* = 4). **(C)** CB1R signal intensity in different amygdala nuclei: lateral amygdala nucleus (LA), basolateral amygdala nucleus (BLA), basomedial amygdala nucleus (BMA), central amygdala nucleus (CeA), and medial amygdala nucleus (MeA). **(D)** Correlation between CB1R immunostaining and maximum limbic seizure severity in different amygdala nuclei. Data are expressed by mean ± standard error mean (SEM). Equal letters represent significant differences (*p* < 0.05) between groups: “a” in comparison to WAR; “b” in comparison to WAR-NLR. Scale bar: 500 μm. Color code scale (8 bits image): 0–255 (min–max).

Similar as we did in the hippocampus, we verified if the AuK induced differences in CB1R expression in the amygdala of WAR LR and WAR NLR. After the AuK, CB1R immunostaining was increased in the total amygdala area of WARs NLR, when compared to control WARs and WARs LR (*p* < 0.05) (Figures 6A,B).

To verify if the changes observed in CB1R immunostaining between recruited and non-recruited WARs were associated with specific amygdala subregions, different amygdala nuclei were analyzed. We observed that WARs NLR showed increased CB1R immunostaining in all amygdaloid areas analyzed in comparison to control WARs (*p* < 0.05). Although in all amygdala structure analyzed the mean of CB1R intensity was higher in the WAR LR in comparison to control WARs, no significant difference was observed. Moreover, differences between recruited and non-recruited WARs were observed in specific regions. CB1R immunostaining was increased in the LA, BLA, BMA, and CeA (*p* < 0.05), but not in the MeA, of non-recruited WARs, when compared to recruited. See Figure 6C.

Finally, we observed a correlation between CB1R immunostaining and the maximum seizure severity. The correlation was observed only in the amygdala and was exclusively associated to limbic seizure severity. The correlation indicates that CB1R immunostaining decreases with the limbic seizure severity. These correlations were observed only in the LA, BLA, and BMA amygdala nuclei (Figure 6D).

## Discussion

In the present study, we observed increased CB1R immunostaining in specific limbic brain regions of WARs, a genetic model of epilepsy, when compared to control Wistar rats. Specifically, WARs showed increased endogenous CB1R immunostaining in the iMO layer of the dorsal hippocampus in comparison to Wistars. Additionally, we showed that both acute and chronic AS in WARs were capable of increasing CB1R immunostaining in several layers of the dorsal hippocampus, as well as in the amygdala. It is worth to mention that during the data analysis we observed intense CB1R immunostaining in many other brain sites such as hypothalamus, thalamus, and several cortical areas, like the piriform and motor cortices (Supplementary Figure 1). However, since our objective in the present study was to analyze CB1R immunostaining in limbic structures (hippocampus and amygdala) important to limbic seizure expression during the AuK, these data about CB1R expression in extra-limbic structures will not be discussed.

Previous studies have shown changes in CB1R expression in limbic brain structures in preclinical models of seizures and humans with TLE. Wallace et al. (2003) showed increased eCBs levels and CB1R expression in the hippocampus of rats submitted to the pilocarpine-induced *Status Epilepticus (SE)* model (Cavalheiro et al., 1991). Moreover, time-dependent effects on CB1R expression were also observed in the pilocarpine model of *SE*. Specifically, hippocampal CB1R expression was reduced in Sprague-Dawley rats during the acute phase, 4 days after pilocarpine injection, while CB1R expression increased in the chronic phase, 4 months after pilocarpine (Falenski et al., 2007, 2009). Similar results were observed in mice that developed severe seizures (Racine's scale 5) after pilocarpine injection. CB1R neuroplastic changes were observed throughout the hippocampus, although CB1R immunostaining was too intense to distinguish hippocampal layers (Karlócai et al., 2011). Additionally, Karlócai et al. (2011) described a high increase of CB1R in the hippocampal SP layer, similarly as we showed in the present study after acute and chronic AS throughout the entire Cornu Ammonis area. Maglóczky et al. (2010) observed increased CB1R expression in hippocampal GABAergic terminals in tissues from sclerotic human brain and from mice submitted to the pilocarpine-induced *SE*. Considerable increase on CB1R expression was observed especially in the molecular layer of the dentate gyrus (Maglóczky et al., 2010). The molecular layer of the dentate gyrus is an important region in the present study, not only because of the increased CB1R immunostaining in both sMO and iMO after acute and chronic AS, but also due to the endogenous increased expression observed in non-stimulated WARs in comparison to Wistars.

The main feature of the AuK is the forebrain and cortical recruitment associated with the development of behavioral clonic and limbic seizures in genetic susceptible animals (Marescaux et al., 1987; Naritoku et al., 1992; Romcy-Pereira and Garcia-Cairasco, 2003; Vinogradova, 2017). As we showed in the present study, during the AuK, the initially brainstem-dependent seizures, give rise to limbic seizures dependent on forebrain and limbic structures, like amygdala, cortex, and hippocampus (Moraes et al., 2000; Romcy-Pereira and Garcia-Cairasco, 2003; Galvis-Alonso et al., 2004; Poletaeva et al., 2017). Additionally, several behavioral and physiological changes are associated with seizure susceptibility and neuropsychiatric comorbidities in WARs and other audiogenic rodent strains (Castro et al., 2017; Garcia-Cairasco et al., 2017; Poletaeva et al., 2017; Aguilar et al., 2018). Furthermore, reduced GABAergic currents were observed in CA1 pyramidal neurons of WARs (Cunha et al., 2018) and volumetric increase was detected in limbic structures of WARs, like the dorsal hippocampus and amygdala, when compared to Wistars rats (Lee et al., 2018). These data indicate functional and anatomical alterations associated with epilepsies in WAR's limbic brain network.

Historically, before the genetic selection of the WARs, we had 10% susceptible Wistars, which became the parentals (founders) of the WARs strain (Garcia-Cairasco et al., 2017). Additionally, it is common to submit Wistar rats to chronic acoustic stimulation as a control for kindling in WARs and eliminate from the study those Wistars which eventually present audiogenic responses. Therefore, Wistar resistant rats submitted to the AuK are named as chronically stimulated (not kindled), because kindled animals mean those with behavioral and EEG seizures (Garcia-Cairasco et al., 2017). Studies with synchronized video-EEG, observed behavioral seizures concomitant to epileptic-like activity in the amygdala, hippocampus and cortex of WARs after the AuK protocol, but Wistars did not develop any EEG alteration in these forebrain structures after chronic acoustic stimulation (Moraes et al., 2000; Romcy-Pereira and Garcia-Cairasco, 2003). Moreover, it is worth noting that AuK induced a decrease in WAR's spatial memory retention in the Morris water maze test, but the chronic acoustic stimulation (no seizures) had no effect on Wistar's performance in the same memory test. Moreover, neurotransmission in the hippocampal Schaffer-collaterals fibers and their excitability were not affected by the chronic exposure to acoustic stimulation in Wistar rats (Cunha et al., 2015) and AuK-dependent limbic recruitment in WARs is not associated with any inflammatory process or oxidative stress, suggesting that this neuroplastic anatomical change is a “network expansion process,” primarily linked to the genetic selection for the epilepsy phenotype in WARs (de Deus et al., 2020). Therefore, except for the small percentage of Wistar susceptible animals (usually discarded), acoustic stimulation does not induce audiogenic seizures in Wistar rats, as well as epileptic-like alterations or epilepsy-related comorbidities.

In the present study, we showed increased CB1R immunostaining in several hippocampal regions, including the SP layer throughout the entire Cornu Ammonis area (CA1–CA3), where hippocampal pyramidal neurons are located. Therefore, it is possible that changes in CB1R expression are related to deficits in GABA currents previously described in the hippocampus of WARs (Cunha et al., 2018). We know that CB1R agonist administration decreases GABAergic activity in hippocampal CA1 area and in the BLA (Hoffman and Lupica, 2000; Katona et al., 2001; Wilson and Nicoll, 2001). Additionally, cannabidiol (CBD) treatment for epilepsy protected animals from seizures and reduced hippocampal hyperexcitability increasing GABAergic currents (Kaplan et al., 2017). However, the direct relationship between CB1R and alterations in hippocampal GABA release observed in WARs still needs to be assessed.

Pharmacological manipulations of CB1R were associated with anticonvulsant effects in several models of epileptic seizures, but only a few studies assessed the role of CB1R in audiogenic models (Rosenberg et al., 2017; Lazarini-Lopes et al., 2020). Systemic activation of CB1R receptors presented protective effects against audiogenic seizures in the Krushinsky-Molodkina strain and in DBA/2 mice (Vinogradova and Van Rijn, 2015; Citraro et al., 2016). Conversely, antagonism of CB1R facilitated the appearance of clonic behavior during the AuK and induced the reappearance of seizures in animals that had previously developed resistance to audiogenic seizures (Vinogradova et al., 2011). Moreover, in WAG/Rij rats, a genetic model of absence seizures, with a subpopulation also susceptible to audiogenic seizures (Vinogradova, 2008), pharmacological activation of CB1R attenuated absence seizures, but the effects on AS were not assessed (Van Rijn et al., 2010; Citraro et al., 2013).

The ECS is a complex system capable of modulating many other mechanisms, for these reason we should not discard possible involvement of CB1R with glutamatergic neurons and/or astrocytes signaling (Monory et al., 2006; Maroso et al., 2016; Busquets-Garcia et al., 2018), especially because overexpression of glutamate receptors subunits have already been observed in the hippocampus of WARs after acute and chronic AS (Gitaí et al., 2010). Therefore, investigating not only the expression, but also the location of CB1R in specific cell types in genetic models of epilepsies can bring important information regarding the ECS and seizure control. Finally, other receptors related to endocannabinoid signaling, such as the transient receptor potential vanilloid 1 (TRPV1) and the GPR55, both activated by anandamide and associated with anticonvulsant effects (Van Der Stelt et al., 2005; Ryberg et al., 2007; Lazarini-Lopes et al., 2020), could be partially involved with CB1R modulation and should be further investigated in the genetic models of epilepsies.

Our results of increased CB1R expression in limbic brain regions are in agreement with other animal models and humans with TLE (Wallace et al., 2003; Falenski et al., 2007; Maglóczky et al., 2010; Karlócai et al., 2011; Rocha et al., 2020). We observed increased endogenous CB1R expression in WARs in comparison to Wistar rats in the iMO of the hippocampus. Alterations in the iMO, such as increased granule cell dendrites, increased endings of apical dendrites, and increased neo-Timm staining, have already been described after pilocarpine-induced *SE* (Arisi and Garcia-Cairasco, 2007). Our results bring new insights about ECS alterations in the molecular layer of the hippocampus, reinforcing the role of this brain region in seizure susceptibility and control. Moreover, we suggest that endogenous increase of CB1R in the iMO of WARs could be associated with the genetic susceptibility to audiogenic seizures and the LR consequent to AuK.

Additionally, we bring important information regarding acute brainstem seizures and modulation of CB1R expression in limbic brain regions. Strikingly, CB1R was increased in hippocampus and amygdala after acute and chronic brainstem seizures. Our results suggest that in audiogenic models of seizures, brainstem seizures, either acute or chronic, are capable of modulating CB1R expression in a similar manner, increasing its expression in the amygdala and hippocampus. Additionally, differences in CB1R expression between WAR NLR and WAR LR were observed in the amygdala (LA, BLA, BMA, CeA), but not in the hippocampus. Also, a decrease of CB1R expression in the amygdala (LA, BLA, BMA) was correlated with increased limbic seizure severity. Curiously, these amygdala subnuclei are the main amygdaloid areas related to limbic recruitment in WARs and in other audiogenic strains, with histological and EEG alterations (Moraes et al., 2000; Galvis-Alonso et al., 2004; Tupal and Faingold, 2010). Therefore, these results suggest that the ECS could be associated with the epileptogenic process in WARs. Nonetheless, the functional consequences of these differences in CB1R expression still need to be investigated, especially because it is unclear if changes in CB1R expression were previously present in LR and NLR WARs before the beginning of the AuK. Additionally, the analysis of CB1R in different time-points during chronic seizures can bring important information about the progressive alterations in CB1R expression during chronic seizures.

The present study has some methodological limitations, such as the absence of EEG recordings in WARs, the lack of analysis of the CB1R functionality or the lack of evaluation of other ECS components. In addition to the assessment of CB1R in the current WAR brain sites (hippocampus and amygdala), directly related to limbic seizure generation, it would be interesting and necessary in the future, to evaluate the expression of CB1R at the sensory processing level, initially the auditory receptor and lower brainstem auditory structures, as well as nuclei of sensory-motor integration systems, the outcome for the manifestation of motor seizures (see details in Garcia-Cairasco et al., 2017). It is important to note that behavioral and immunohistochemical data were consistent in the present study and we showed the first evidence of endogenous changes in CB1R expression in WARs, a genetic model of epilepsy, which is able to mimick tonic-clonic seizures (acute protocol) and TLE (chronic protocol, kindling). It is important to note that we used a genetic model of epilepsy with no need of any chemical of electrical stimulation to induce seizures in WARs. Due to these features, genetic models of epilepsies with reflex seizures, like audiogenic strains, are important methodological approaches in the preclinical epilepsy research (Faingold et al., 2014; Garcia-Cairasco et al., 2017). Moreover, we applied a detailed anatomical analysis, which allowed the assessment of CB1R expression in specific brain regions and subregions. Only using this exhaustive and accurate methodology, it was possible to detect the changes in the iMO of the hippocampus of control WARs, as well as to identify specific regions of the amygdala where CB1R expression was correlated to limbic seizure severity.

Therefore, we conclude that WARs present increased endogenous CB1R expression in the iMO, when compared to control Wistar rats. Additionally, acute and chronic (kindled) audiogenic seizures increased CB1R expression in several hippocampal and amygdala regions. Furthermore, changes in CB1R expression in the amygdala, but not in the hippocampus, were correlated with limbic seizure severity in WARs. Our study is the first to characterize CB1R in the WAR strain, a genetic model of epilepsy with brainstem (acute protocol) and limbic (chronic protocol) seizures. Our results agree with previous studies, supporting that changes in the ECS may be related with the seizure susceptibility and its control. Further studies investigating ECS components in genetic models of epilepsies, such as WAR, GEPR, and WAG/Rij, may bring important information about seizure susceptibility and pharmacological control. Future mapping studies would also expand the search for brain sites of expression of CB1R, associated to epilepsies and epilepsy-related comorbidities.

## Data Availability Statement

The raw data supporting the conclusions of this article will be made available by the authors, without undue reservation.

## Ethics Statement

The animal study was reviewed and approved by Ethics Committee in Animal Research of the Ribeirão Preto School of Medicine at the University of São Paulo (Protocol number: 057/2017).

## Author Contributions

WL-L, RV-S, RS-J, and NG-C conceived the original idea. WL-L conducted behavioral and immunohistochemical experiments, imaging protocols, and analysis. RV-S collected brain tissue. RS-J and GS-M performed immunohistochemical analysis, image processing, and prepared figures. WL-L prepared figures and wrote the manuscript. NG-C made important intellectual contributions and obtained funding. All authors reviewed, discussed, and approved the manuscript.

## Conflict of Interest

The authors declare that the research was conducted in the absence of any commercial or financial relationships that could be construed as a potential conflict of interest.

## Supplementary Material

Supplementary Figure 1 Representative images of CB1R immunostaining in extra-limbic brain structures. Different experimental groups (left column) and their correspondent heatmap (right column) of CB1R immunostaining. It is possible to observe intense CB1R immunostaining in several brain areas, besides the hippocampus and amygdala, such as the hypothalamus, the thalamus, and several cortical areas. Scale bar: 500 μm. Color code scale (8 bits image): 0–255 (min–max).

Supplementary Table 1 Number of animals and sections used to analyze CB1R immunostaining in each experimental group.
