# Supplementary figures and images for: Cannabinoid Receptor Type 1 (CB1R) Expression in Limbic Brain Structures After Acute and Chronic Seizures in a Genetic Model of Epilepsy
Source: Front Behav Neurosci. 2020 Dec 21;14:602258. doi: 10.3389/fnbeh.2020.602258 (PMC7779524; doi:10.3389/fnbeh.2020.602258)

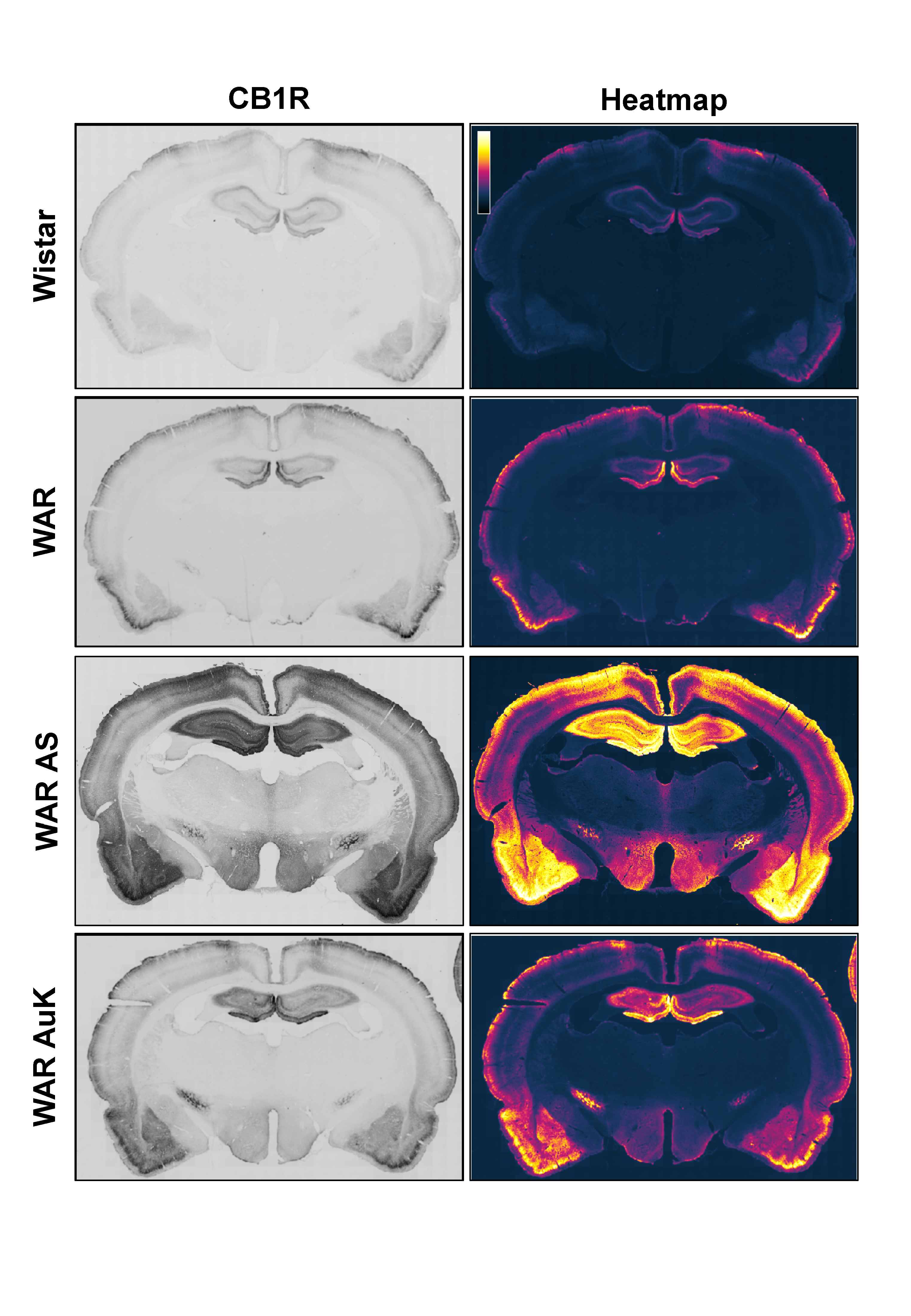

Supplement: Supplementary Figure 1 — Representative images of CB1R immunostaining in extra-limbic brain structures. Different experimental groups (left column) and their correspondent heatmap (right column) of CB1R immunostaining. It is possible to observe intense CB1R immunostaining in several brain areas, besides the hippocampus and amygdala, such as the hypothalamus, the thalamus, and several cortical areas. Scale bar: 500 μm. Color code scale (8 bits image): 0–255 (min–max). [file Image_1.tif]
