# Supplementary material for: Cannabinoid Receptor Type 1 (CB1R) Expression in Limbic Brain Structures After Acute and Chronic Seizures in a Genetic Model of Epilepsy
Source: Front Behav Neurosci. 2020 Dec 21;14:602258. doi: 10.3389/fnbeh.2020.602258 (PMC7779524; doi:10.3389/fnbeh.2020.602258)
Supplement: Supplementary Table 1 — Number of animals and sections used to analyze CB1R immunostaining in each experimental group. [file Table_1.docx]

| **Exp. Group** | | **Total Area of the Dorsal Hippocampal** | | | **Hippocampal Layers** | | | **Hippocampal *Cornu Ammonis* Area (CA1, CA2, and CA3)** | | | **Total Area of the Amygdala** | | | **Amygdala**  **Subnuclei** | | |
| --- | --- | --- | --- | --- | --- | --- | --- | --- | --- | --- | --- | --- | --- | --- | --- | --- |
|  |  |  | **Rat ID** | **Sections Analyzed** |  | **Rat ID** | **Sections Analyzed** |  | **Rat ID** | **Sections Analyzed** |  | **Rat ID** | **Sections Analyzed** |  | **Rat ID** | **Sections Analyzed** |
| **WAR AuK** | **AuK-LR** | N=6 | 1 | 5 | N=6 | 1 | 10 | N=6 | 1 | 7 | N=6 | 1 | 9 | N=6 | 1 | 9 |
|  |  |  | 2 | 5 |  | 2 | 5 |  | 2 | 5 |  | 2 | 4 |  | 2 | 4 |
|  |  |  | 3 | 4 |  | 3 | 7 |  | 3 | 4 |  | 3 | 6 |  | 3 | 6 |
|  |  |  | 4 | 10 |  | 4 | 12 |  | 4 | 8 |  | 4 | 5 |  | 4 | 5 |
|  |  |  | 5 | 5 |  | 5 | 5 |  | 5 | 5 |  | 5 | 5 |  | 5 | 5 |
|  |  |  | 6 | 5 |  | 6 | 5 |  | 6 | 4 |  | 6 | 7 |  | 6 | 7 |
|  | **AuK-NLR** | N=4 | 1 | 6 | N=4 | 1 | 6 | N=4 | 1 | 5 | N=4 | 1 | 5 | N=4 | 1 | 5 |
|  |  |  | 2 | 6 |  | 2 | 6 |  | 2 | 6 |  | 2 | 7 |  | 2 | 7 |
|  |  |  | 3 | 10 |  | 3 | 10 |  | 3 | 8 |  | 3 | 7 |  | 3 | 7 |
|  |  |  | 4 | 6 |  | 4 | 6 |  | 4 | 4 |  | 4 | 4 |  | 4 | 4 |
| **WAR AS** | | N=3 | 1 | 7 | N=5 | 1 | 8 |  | | | N=5 | 1 | 8 | N=5 | 1 | 8 |
|  |  |  | 2 | 5 |  | 2 | 6 |  |  |  |  | 2 | 7 |  | 2 | 7 |
|  |  |  | 3 | 6 |  | 3 | 10 |  |  |  |  | 3 | 6 |  | 3 | 6 |
|  |  |  |  | |  | 4 | 9 |  |  |  |  | 4 | 8 |  | 4 | 8 |
|  |  |  |  |  |  | 5 | 5 |  |  |  |  | 5 | 7 |  | 5 | 7 |
| **WAR** | | N=5 | 1 | 6 | N=5 | 1 | 14 | N=5 | 1 | 6 | N=5 | 1 | 7 | N=5 | 1 | 7 |
|  |  |  | 2 | 6 |  | 2 | 10 |  | 2 | 5 |  | 2 | 10 |  | 2 | 10 |
|  |  |  | 3 | 3 |  | 3 | 6 |  | 3 | 5 |  | 3 | 8 |  | 3 | 8 |
|  |  |  | 4 | 2 |  | 4 | 8 |  | 4 | 4 |  | 4 | 5 |  | 4 | 5 |
|  |  |  | 5 | 3 |  | 5 | 11 |  | 5 | 6 |  | 5 | 4 |  | 5 | 4 |
| **Wistar** | | N=5 | 1 | 4 | N=5 | 1 | 4 |  | | | N=5 | 1 | 6 | N=5 | 1 | 6 |
|  |  |  | 2 | 7 |  | 2 | 8 |  |  |  |  | 2 | 4 |  | 2 | 4 |
|  |  |  | 3 | 5 |  | 3 | 10 |  |  |  |  | 3 | 7 |  | 3 | 7 |
|  |  |  | 4 | 6 |  | 4 | 8 |  |  |  |  | 4 | 8 |  | 4 | 8 |
|  |  |  | 5 | 6 |  | 5 | 10 |  |  |  |  | 5 | 8 |  | 5 | 8 |
